# Effectiveness of wheelchair skills training for improving manual wheelchair mobility in children and adolescents: protocol for a multicenter randomized waitlist-controlled trial

**DOI:** 10.1186/s12887-023-04303-8

**Published:** 2023-09-26

**Authors:** K. L. Best, P. W. Rushton, J. Sheriko, K. P. Arbour-Nicitopoulos, T. Dib, R. L. Kirby, M. E. Lamontagne, S. A. Moore, B. Ouellet, F. Routhier

**Affiliations:** 1 School of Rehabilitation Sciences, Faculty of Medicine, Université Laval, Quebec City, QC G1V 0A6 Canada; 2 Center for Interdisciplinary Research in Rehabilitation and Social Integration, Centre Intégré Universitaire de Santé Et de Services Sociaux de La Capitale-Nationale, 525 Wilfrid-Hamel Boulevard, Quebec City, QC G1M 2S8 Canada; 3 School of Rehabilitation, Université de Montréal, Montréal, QC Canada; 4 CHU Sainte-Justine Research Center, Marie Enfant Rehabilitation, Montréal, QC H1T 1C9 Canada; 5 School of Health and Human Performance, Faculty of Health, Dalhousie University, Halifax, NS B3H 4R2 Canada; 6 Department of Pediatrics, Faculty of Medicine, Dalhousie University, Halifax, NS B3H 4R2 Canada; 7 Faculty of Kinesiology and Physical Education, University of Toronto, Toronto, ON M5S 2W6 Canada; 8 Division of Physical Medicine and Rehabilitation, Dalhousie University, Halifax, NS B3H 4K4 Canada

**Keywords:** Children, Adolescents, Youth, Disability, Manual wheelchair, Wheelchair skills training, Participation

## Abstract

**Background:**

Self-directed mobility during childhood can influence development, social participation, and independent living later in life. For children who experience challenges with walking, manual wheelchairs (MWCs) provide a means for self-directed mobility. An effective MWC skills training program exists for adults, but controlled trials have not yet been documented in children and adolescents. This paper outlines the protocol for a multi-centre randomized wait-list controlled trial. The primary objective is to test the hypothesis that children and adolescents who receive MWC skills training will have higher MWC skills capacity compared to children and adolescents in the control group who receive usual care. The secondary objectives are to explore the influence of MWC skills training in children and adolescents (MWC use self-efficacy and satisfaction with participation in meaningful activities), and parents (perceived MWC skills); and to measure retention three months later.

**Methods:**

A multi-centre, parallel-group, single-blind randomized wait-list controlled trial will be conducted. A sample of 60 children and adolescents who use MWCs will be recruited in rehabilitation centres, specialized schools, and the communities of three Canadian cities. Participants will be randomized (1:1) to the experimental (Wheelchair Skills Training Program [WSTP]) or wait-list control group (usual care). Performance-based and self-report measures will be completed at baseline (T1), three months (post-intervention, T2), and three months post-intervention (T3). The primary outcome will be MWC skills capacity post-intervention. Secondary outcomes will be MWC use self-efficacy and satisfaction with participation of the child/adolescent, and parent-perceived MWC skills. The WSTP will consist of 12 sessions, 45–60 min each, delivered 1–2 times per week by trained personnel with health professions education. Training will be customized according to the child’s baseline skills and participation goals that require the use of the MWC. The wait-list control group will receive usual care for 3 months and then receive the WSTP after completing T2 evaluations. Data will be analysed using ANCOVA (controlling for baseline scores).

**Discussion:**

MWC skills training may be one way to improve self-directed mobility and related outcomes for children and adolescents. The results of this multi-centre randomized wait-list controlled trial will allow for the effectiveness of the intervention to be evaluated in a variety of clinical contexts and geographical regions.

**Trial registration:**

ClinicalTrials.gov: NCT05564247, Version October 3, 2022.

## Background

Self-directed mobility during childhood, defined as the ability to move in the manner and at the time of one’s own choice [1], is associated with attainment of developmental milestones, social participation, and increased likelihood of gainful employment and independent living in adulthood [2–4]. For children and adolescents who have trouble walking, manual wheelchairs (MWC) may be helpful. However, children and adolescents who use MWCs often rely on their parents and others for mobility, and thus may miss opportunities for participation, and play [5]. Moreover, they may have fewer opportunities to make choices and decisions about what they want to do and where they want to go, which may contribute to learned helplessness, restricted participation in physical activities, and social isolation [6–12]. Although self-directed mobility can contribute to child development and positively influence health-related quality of life and self-esteem [13–16], simply providing a MWC does not guarantee independent or safe use. The World Health Organization recommends a four-step approach to wheelchair service provision, one of which is training [1].

Improper MWC use can increase the risk of acute injuries due to accidents (e.g., tips, falls) and repetitive strain injuries [17, 18]. Between 1991 to 2008, approximately 44,300 American children and adolescents were treated in emergency departments for injuries related to using a wheelchair [19]. Furthermore, inadequate MWC skills can limit mobility and restrict participation, thus increasing risk of secondary chronic health conditions (e.g., cardiovascular disease, diabetes, obesity) [20]. In fact, MWC skills competence was highlighted as an important factor when aiming to improve participation in physical activity, which promotes health and quality of life [21].

Despite the importance of MWC skills, few children and adolescents receive adequate training to improve the skills needed for safe, self-directed mobility in the community and social participation. For example, in a survey of 68 Canadian rehabilitation centres (43 of which provided services to children), more than half reported that therapists trained MWC skills for two hours or less while 18% of centres reported therapists provided no training at all [22]. Similarly in one Canadian rehabilitation centre, 18 parents and child and adolescent MWC users affirmed receiving less than adequate MWC skills training [23]. In this study, 64% of occupational therapists from two specialized schools and a rehabilitation centre for children and adolescents reported they provided MWC skills training, but only 4% did so as a part of their regular practice. In the total sample, 26% used a standardized program such as the Wheelchair Skills Training Program (WSTP), and 74% spent an average of 3 h or less on training [23]. In both studies, the key barriers to providing MWC skills training were lack of time, resources, and knowledge [22, 23].

The WSTP, the training component of the evidence-based Wheelchair Skills Program (WSP), may help to address training needs for children and adolescents, as it is safe and effective for improving MWC skills in various adult populations (e.g., wheelchair users, therapists, caregivers) and in a variety of rehabilitation and community settings [24, 25]. The evidence supporting the WSTP approach in children and adolescents is promising, with four single-group studies (total *n*= 28) showing statistically significant within-subject improvements in MWC skills after training [26–29]. Training with children and adolescents was conducted in clinical and community settings, including as part of regular physical education classes during the academic year [27], and during two condensed 2–4 h sessions [26, 28]. Parents also perceived that their child had increased independence, improved MWC skills, and decreased shoulder pain after completing the WSP [26, 28]. However, to date no randomized controlled trials have been documented in children and adolescents.

This paper outlines the protocol of a randomized wait-list controlled trial which aims to document the influence of the WSTP on MWC skills and mobility outcomes in children and adolescents. The primary objective is to test the hypothesis that children and adolescents who receive the WSTP will have higher MWC skills capacity compared to children and adolescents in the control group who receive usual care. The secondary objectives are to explore: 1) the effects of the WSTP on MWC activity, MWC use self-efficacy and satisfaction with participation in meaningful activities among children and adolescents; 2) the influence of the WSTP on parent-perceived MWC skills capacity and performance and satisfaction with participation; and 3) retention of all primary and secondary outcomes among children, adolescents, and parents 3-months post-intervention.

## Methods

### Design

A multi-site, parallel-groups, single-blind (testers) randomized wait-list controlled trial will be conducted in three Canadian cities (Quebec City, Montreal, Halifax). Participants will be randomly assigned to the experimental group (WSTP) or the control group (usual care) using a 1:1 allocation ratio between groups. A computerized randomization process, stratified by site (3 sites) and age (categorized according to the National Institute of Child Health and Human Development, which defines childhood as 2 to 11 years and adolescence as 12 to 18 years of age [30]) with undisclosed block sizes will be conducted by a research professional who is not involved in the study, and the allocation sequence will be placed in sealed opaque envelopes. A trial coordinator at the primary site (Quebec City) who is not involved in any other aspect of the research will manage randomization for all sites. The site coordinator will email the trial coordinator after completion of T_1_ assessments, and the trial coordinator will open the sealed envelope and reply with participant allocation within 24 hours. A site coordinator at each site will communicate with the trial coordinator and will manage recruitment, enrollment, and participant flow through the study.

### Participants and recruitment

To be included in this study, children and adolescents will be between the ages of 4 to 18 years (based on the National Institute of Child Health and Human Development definition [30] and education and rehabilitation services in Eastern Canada); have a MWC for mobility purposes; be able to self-propel a MWC for at least 10 m; be willing to participate in the study (create participation goals at baseline); be able to indicate a need or answer a simple question (e.g., multiple choice) verbally or with a non-oral mode of communication (e.g., pictograms, communication devices); and be able to follow a two-step command evaluated in one of two ways: 1) the parent or guardian who completes eligibility screening is asked by the research coordinator whether their child can follow a two-step command or 2) the clinician involved in recruitment is asked by the research coordinator whether their child can follow a two-step command. Exclusion criteria include children who use a MWC with double hand-rim (as the WSTP does not currently include specific training instructions), an anticipated health condition or procedure that could contraindicate training or testing in the next six months (e.g., surgery); and a degenerative condition that is expected to progress quickly.

Convenience sampling will be used to recruit 60 children and adolescents (targeting 20 per site), a sample size based on a power analysis (described below). Active recruitment strategies will include working with clinical collaborators to recruit in local rehabilitation centres and specialized schools in Quebec City (Centre intégré universitaire de santé et de services sociaux de la Capitale-Nationale (CIUSSS-CN), Madeleine Bergeron), Montreal (Marie-Enfant Rehabilitation Centre (CRME), Victor Doré, Joseph Charbonneau, Jean-Piaget) and Halifax (IWK Health Centre, Westmount). Clinicians from the pediatric rehabilitation centres at each site will discuss the study with families who may be eligible. Passive recruitment strategies will also include dissemination of promotional videos and posters on social media and websites of collaborators and community organizations.

### Procedures

**Fig. 1:**
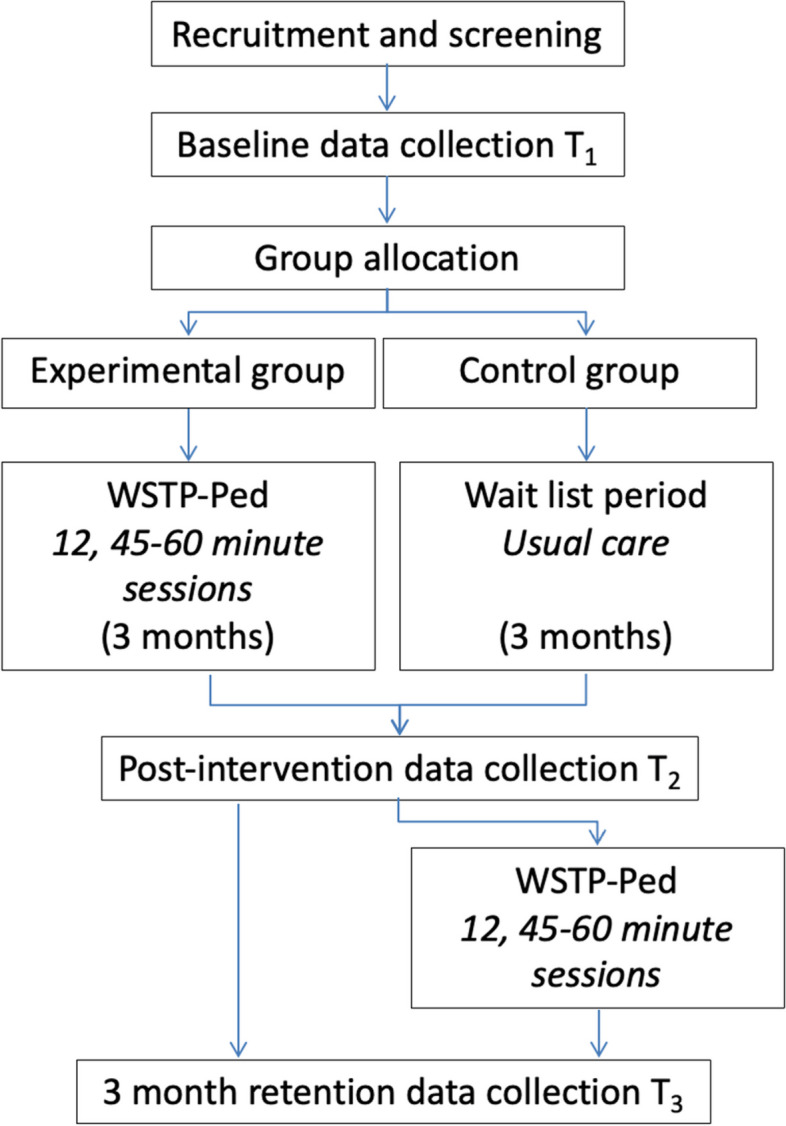
Trial design describing coordination, testing and experimental procedures

Participants will complete baseline measures (T_1_) and then be assigned to the experimental or control group. The site coordinator will contact the trainer after T_1_ for participants assigned to the intervention group_._ Participants will be asked to attend 12 sessions of the WSTP intervention or continue to receive usual care (3 months), and then complete post-intervention outcomes (T_2_), ideally within a target of one to four days after training. All outcomes will be collected three months after T_2_ to assess retention of skills (T_3_). The site coordinator will schedule all assessments at T_1,_ T_2_ and T_3_ for both groups, as testers will remain blinded to group assignment throughout the study. The site coordinator will call participants 24–48 h in advance of testing and remind the participants about the importance of not revealing to the tester in which group they were assigned. Tester training will include watching an instructional video series (1 h, 37 min total) on how to complete the outcomes and customized in-person or virtual training depending on experience and needs. All data will be stored in a locked channel on Microsoft teams that will be only accessible to testers and the primary investigators. The trial design is depicted in Fig. 1.

### Intervention (WSTP)

Participants allocated to the experimental group will complete a target of 12, 45–60 min sessions of the WSTP at a target frequency of 1 to 2 sessions per week. The WSTP will be delivered by trained personnel with education or experience in relevant fields (e.g., occupational therapy, physiotherapy, kinesiology, recreation therapy, adapted physical activity). Trainers may have various combinations of clinical practice or research experience with the WSTP. All trainers will complete self-directed WSTP training modules and hands-on practice until readiness to provide training is confirmed by Best, Rushton or Kirby (each have more than 20 years of experience in training individuals to administer the WSTP).

According to the WSP lesson plan [31], trainers will customize each session based on the age group (child or adolescent) and training goals of each child as determined during baseline testing and in the first training session. Goals can be adapted and modified over the course of the 12 weeks, which may increase motivation and adherence to the training protocol. Subsequent sessions will begin with a 5-min review of progress and socializing, followed by a 10-min warm-up (random practice of the previously learned skills); 20-min of attempting new skills (training on each skill will be carried forward to the next session until the skills are learned or until the trainer and participant mutually agree that training should be abandoned; the trainer will periodically ask the participant to practice newly learned skills to incorporate variability of practice); 10-min cool-down, during which the participant will practice skills in a self-determined manner.

The intervention will be delivered at university-affiliated health or research centres and the surrounding community (e.g., schools, homes, libraries, museums, parks) depending on preferred location and the child’s goals to enhance transferability of skills to real-life settings and to improve adherence to the intervention [31]. Fidelity of the WSTP administration will be evaluated by a member of the study team not involved in testing or training, who will watch one training session per participant (either in person or by video) randomly and at various times throughout the training period. The trainer will also complete a detailed training log and a checklist of standardized steps to be followed during training. Feedback and monthly discussions with trainers at each site will enhance fidelity. Any adverse events will be documented in the training logbook by the trainer. The logbook will also be used to document whether the participant requests to stop training or if they experience any health conditions that may impact or stop the training.

### Wait-list control group

Children and adolescents in the control group will receive usual care during a 3-month period, which varies between and within regions [22]. During this time, participants will be considered on a waitlist to receive WSTP training and can continue or start any activities provided by therapists or in the community. After completion of T_2_ evaluations, children and adolescents in the control group will complete a target of 12, 45–60 min sessions of the WSTP, delivered by trained personnel as described for the experimental group.

Acknowledging that usual care may include some form of MWC skills training, previous evidence suggests the amount of training received during usual care according to the WSTP will be limited [22, 23].

### Data collection methods

Sociodemographic and wheelchair data (age, gender, diagnosis, previous MWC use, previous MWC skills training, physical activity, MWC size, frame type, wheel size, add-ons) will be collected from the child and parent (or guardian) at baseline (T_1_).

### Primary outcome

MWC skills capacity (i.e., what a person can do in a standardized environment) [32] will be assessed using the WST Version 5.3.1 for MWC users [31]. The child will be asked to execute 30 MWC skills in a standardized environment. Any adverse events during testing will be documented in the WST data collection form by the tester. Participants will be scored on a 4-point scale as Advanced pass (3), Pass (2), Partial pass (1), Fail (0), No part (NP), and Testing error (TE). The total percentage WST score is calculated as: Total percentage WST Capacity Score = sum of individual skill scores / ([# of possible skills – # of NP scores – # of TE scores] × 3) X 100%. As of February 8, 2023, there have been 112 studies indexed in PubMed about the WST (or the questionnaire version [WST-Q]) or that have used these as outcome measures [31]. A minimally clinical important difference (MCID) of 20% relative improvement in WST scores (T_1_ to T_2_) has been suggested for adults [24], but has not yet been determined for children.

### Secondary outcomes

Parent/guardian perception of child’s MWC skills level will be measured using the Wheelchair Skills Level Questionnaire, a screening tool based on the WSP sets of skills [31]. A parent (either mother or father) or guardian will respond to one, two or three questions to determine the skill level: Dependent (0); Indoor (1)—the child is able to use their MWC in the place where they live; Community (2)—the child can independently get their MWC over obstacles in their community; Advanced (3)—the child can use a wheelie to overcome obstacles in their community. If the response to a threshold question is “yes”, the child achieves the level, and the evaluator will ask the question of the next level above. If the answer to the next question is “no”, the child remains in the level below (except if the first question was about advanced skills).

Parent/guardian perception of child’s MWC skills performance (i.e., what their child does in their daily life) [32] and frequency (i.e., how often the child performs the skill) will be assessed using the 30-item Wheelchair Skills Test Questionnaire (WST-Q) Version 5.3.1 for MWC users [31]. A parent (mother or father) or guardian will rate how well and how often their child performs each of the 30 skills on the WST-Q. Assessments with parents/guardians will be done before the child completes the WST to reduce the influence of the objective test. The root performance question asks, “Can your child do (the MWC skill) in their own setting?”. Response options include “yes, very well”, “yes, but not well”, “yes, but with help”, “no” and “no part” with this MWC. For frequency, the root question asks, “How often does your child do (the MWC skill) in their own setting?”. Response options include “always”, “usually”, “rarely”, and “never” and “no part” with this MWC. The total percentage WST-Q scores are calculated in the same way as the WST (defined above). The WST-Q provides an understanding of parent’s perceptions of the change in the child’s skill performance between the baseline and follow-up. The same parent (or guardian) will be asked to complete the questionnaire at T_2_ and T_3_.

MWC use self-efficacy will be evaluated using the Wheelchair Use Confidence Scale for Children using Manual Wheelchairs (WheelCon-M-P), a 33-item self-report scale [33]. Responses are represented through a pictorial Likert type scale with descriptors that range from 0 (pictorial: unhappy face; descriptor: not at all confident) to 4 (pictorial: happy face; descriptor: very confident). Testers will allow the child/adolescent to read through the items on the WheelCon-M-P (the items will be read if needed) and ask the child/adolescent to point to the pictorial that best represents how confident they are that they can do the activity alone and safely. Responses will indicate current level of perceived confidence in 6 areas (i.e., navigate the physical environment in a MWC, perform activities in a MWC, problem solve, advocate for needs, and manage social situations and emotions). Reliability and validity have been documented with previous versions of the WheelCon [22, 34]. The WheelCon-M-P has been validated for use in children and adolescents ages 7–18 years with variable diagnoses (e.g., cerebral palsy, spina bifida) [35].

Satisfaction with participation in meaningful activities will be assessed using the Wheelchair Outcome Measure for Young People (WhOM-YP) [36]. The child/adolescent will create meaningful goals with the tester that require the use of a MWC. The goals may include using their MWC indoor and outdoors at home, at school or in the community. The parent/guardian may be involved in goal setting, but the focus will be on developing goals with the child/adolescent. Ratings of perceived ‘importance’ of the goal (0–10) and ‘satisfaction’ with current performance of this activity (0–10) will be obtained. A mean satisfaction with performance of participation is derived by summing the level of satisfaction for each identified goal and then dividing by the total number of goals. The WhOM-YP has been validated for use in children and adolescents [36].

### Data management

Data with children will be collected using paper forms and will be entered into REDCap (Research Electronic Data Capture, hosted at Université Laval) immediately after testing by the tester. All paper forms will be scanned by the tester and uploaded into a secure and locked channel on Microsoft Teams. Data from the parents will be collected using REDCap by a direct link to an online data entry form. REDCap is a secure, web-based software platform designed to support data capture for research studies. Data quality will be promoted by data verification by a second person who was not involved in testing who will verify the data entry using the raw data forms. Range checks for individual items and total scores will be verified periodically by the principal investigators (KB, PR, JS). The data management procedures will be stored in Microsoft Teams.

### Sample size

The sample size was powered to detect a statistically significant difference of 20% between groups [24], using mean change scores from T_1_ to T_2_ in the primary outcome from Best et al., 2016, which reported a large effect size (Cohen’s d = 1.3) for the WST and a correlation between measures (R^2^) of 0.6 [37]. Based on pilot work, children and adolescents have similar variability in WST capacity scores compared to adults (standard deviation varying from 8 to 20) [26, 27]. However, given the unknown variability associated with age range, a more conservative effect size informed by our pilot work (d = 0.85) and measure correlation (0.60) was chosen. With α = 0.05, and 85% power for between-factors ANCOVA repeated measures design (F-tests) controlling for baseline score (account for function/motor development), a sample size of 18 will be required. Given site variability, each site is powered for primary analysis. G*Power v.3.1.9.2 was used for sample size calculations [38]. Adjusting for a 10% loss to attrition, a total sample size of 60 (20 per site) will be required.

### Analyses

Summary statistics (mean, median, standard deviation, and percentage) will describe the sample (e.g., sex, gender, diagnoses, previous MWC use, previous MWC training). Assumptions for parametric testing will be verified and data will be screened for outliers [39]. Intention-to-treat analysis will be used, and missing data will be treated using joint modelling multiple imputation methods for repeated methods of longitudinal data [40]. To address the primary objective, T_2_ WST capacity scores will be compared between the experimental and control group using analysis of covariance (ANCOVA or non-parametric equivalent). Covariates will be selected based on influence suggested in the literature (i.e., function/MWC motor development as measured by baseline scores, previous MWC use). Variance (R^2^), statistical significance (95% confidence interval), and effect sizes (partial *n*^2^) will be assessed using Sums of Squares methods. Secondary objectives will be evaluated using linear mixed-effect models, which are flexible to explore within-subject mixed/random effects [34].

Post-hoc exploratory analyses will investigate the influence of age (childhood, adolescence), gender, and MWC experience on effect size and practice dosage using linear mixed-effect models. Comparisons between WST change scores for the experimental group (T_1_-T_2_) and WST changes scores after training in the control group (T_2_-T_3_) will be explored using t-tests. Sensitivity analyses will be conducted on demographic variables (e.g., age, sex, gender) to generate hypothesis and inform future investigation.

#### Steering committee

A steering committee will be comprised of a member of the research team, a student member of the research team, a clinical program director of pediatric services related to mobility, an occupational therapist who works with children who use MWCs, a child educator with bioethical training, a parent of a child who uses a wheelchair, a child (14 years +) who uses a MWC. There will be representation from all three cities involved in this trial. A statistician will be consulted by the primary investigator at mid-trial to perform an interim analysis. A decision to stop the trial will be made by co-principal investigators (Best, Rushton, Sheriko) as informed by the statistical consult.

## Discussion

This paper described the protocol for a randomized wait-list controlled trial to evaluate the effect of the WSTP on MWC skills capacity in children and adolescents, and to explore the influence on MWC use self-efficacy, satisfaction with participation and parents’ perceptions of MWC skills.

Three Canadian sites (Quebec City, Montreal, Halifax) represent major urban centres with access to a large population of children and adolescents who use MWCs. The MWC procurement process in Canada is variable, and there are differences in geography, culture, and climate. In the province of Quebec, MWC are covered by the provincial healthcare system, but services focus mainly on MWC selection and set-up. Observationally, MWCs are replaced about every 5 years, with little follow-up on MWC related services, such as training. However, in Nova Scotia (and other Canadian provinces), wheelchairs are commonly purchased through private insurance, by the family, or obtained through wheelchair recycling programs, with limited access to training, especially for those living in rural areas. This diversity will allow the effectiveness of the WSTP to be evaluated in a variety of clinical contexts and geographical regions, for which site-specific post-hoc exploratory analyses will provide additional insights.

Although an attention-matched control group would provide the most rigorous controlled design, pilot work suggested a high risk of attrition and lack of interest to participate in the study. In fact, parents expressed not wanting to risk their child being allocated to a control group if it meant they would not receive MWC skills training. Given the known benefits of the WSTP in adults [24, 25], strong preliminary evidence in children [26–29], and in effort to mitigate attrition and facilitate recruitment, a wait-list control group was justified for this trial. This design will also reinforce clinical equipoise and reduce the risk of bias associated with lack of equipoise.

Enhancing self-directed mobility through MWC skills training may provide children with the dependent, indoor, community and advanced skills needed to use a MWC, thus could enhance participation in activities that require the use of a MWC (e.g., activities at school, sports, and active play), and reduce risks of acute (e.g., tips and falls) and overuse (e.g., repetitive strain due to poor propulsion techniques) injuries. Moreover, clinicians and community organizations have expressed interest in learning about MWC skills evaluation and training for integrating into clinical practice and community programs. For example, a community based physical activity organizing offering adapted physical activity programs would like to offer wheelchair skills training as a weekly program.

Establishing effect size in a randomized controlled trial will provide evidence to support integration of the WSTP into clinical and community partners to implement the WSTP as part of clinical practice and community-based program offering. Facilitators and barriers to using the WSTP may be explored with therapists and community partners. Implementation of the WSTP into regular clinical practice would ensure training starts early, specifically in fundamental skills (e.g., propulsion and maneuvering) and indoor skills required for getting around in the home and school. Extending MWC skills training into the community may reduce burden on rehabilitation therapists while responding to ongoing training needs as the child develops. For example, children receive new MWC as they grow. Approaches to performing community and advanced MWC skills, such as ascending curbs and steep ramp, may vary depending on MWC configuration and set-up. Moreover, having the opportunity to receive training when receiving a new MWC may enhance skills, safety and self-efficacy. In addition, if the child experiences a tip or fall, availability of wheelchair skills training in the community may provide an opportunity to rebuild skills and confidence on an as-needed basis. Future studies may consider implementation evaluation of the WSTP in clinical practice and ecological evaluations when training provided as a community-based program.

### Dissemination plan

A multifaceted knowledge translation strategy aims to increase awareness and knowledge and to change clinical and community practice related to wheelchair skills training and evaluation for children and youth. Targeted promotional and knowledge translation materials will be developed throughout the study for all stakeholders in English and French (children who use MWCs, families, clinicians, community professionals) including books, posters, workbooks, videos and infographics, that will be disseminated using several platforms (e.g., print, social media of clinical and community partners). We will work with community partners to customize end-of-grant promotional and awareness videos for MWC skills training to be shared through social media to prepare for implementation. The WSP and community partner websites will be used to disseminate key messages to researchers, clinicians, and the community. A minimum of three peer-reviewed publications will include a research protocol, results of the main trial, and integrated knowledge translation from clinical RCT to community practice. Results from the study will also be presented at national, international and local conferences.

## Data Availability

Data and materials will be made available upon request to the corresponding author.

## Abbreviations

MWC: Manual wheelchairs
WHO: World Health Organization
WSP: Wheelchair Skills Program
WSTP: Wheelchair Skills Training Program
RCT: Randomized controlled trial
OT: Occupational therapist
WST: Wheelchair Skills Test
NP: Not possible
TE: Testing error
MCID: Minimally clinical important difference
WST-Q: Wheelchair Skills Test Questionnaire
WheelCon-M-P: Wheelchair Use Confidence Scale for Pediatric MWC users
WhOM-YP: Wheelchair Outcome Measure for Young People
ANCOVA: Analysis of covariance
CIUSSS-CN: *Centre intégré universitaire de santé et de services sociaux de la Capitale-Nationale*
CRME: *Centre de réadaptation Marie Enfant*
